# Continued increases in the incidence of healthcare-associated infection (HAI) during the second year of the coronavirus disease 2019 (COVID-19) pandemic

**DOI:** 10.1017/ice.2022.116

**Published:** 2022-05-20

**Authors:** Lindsey M. Lastinger, Carlos R. Alvarez, Aaron Kofman, Rebecca Y. Konnor, David T. Kuhar, Allan Nkwata, Prachi R. Patel, Vaishnavi Pattabiraman, Sunny Y. Xu, Margaret A. Dudeck

**Affiliations:** 1 Division of Healthcare Quality Promotion, Centers for Disease Control and Prevention, Atlanta, Georgia; 2 Leidos, Atlanta, Georgia; 3 CACI, Atlanta, Georgia

## Abstract

Data from the National Healthcare Safety Network were analyzed to assess the impact of COVID-19 on the incidence of healthcare-associated infections (HAI) during 2021. Standardized infection ratios were significantly higher than those during the prepandemic period, particularly during 2021-Q1 and 2021-Q3. The incidence of HAI was elevated during periods of high COVID-19 hospitalizations.

The coronavirus disease 2019 (COVID-19) pandemic has resulted in unprecedented challenges for infection prevention in hospitals. Increases in healthcare-associated infections (HAIs) and device utilization were observed throughout 2020 as hospitals responded to increased patient volumes, increased patient acuity levels and comorbidities, and staffing and supply shortages.^1–4^ To assess for continued impact of COVID-19 on HAI incidence during the second year of the pandemic, we analyzed 2021 data reported to the National Healthcare Safety Network (NHSN) from US acute-care hospitals.

## Methods

This analysis followed the same methodology published for 2020 data.^1^ Quarterly national standardized infection ratios (SIRs) and standardized utilization ratios (SURs) were calculated for the first 3 quarters of 2021 (2021-Q1 through 2021-Q3) for central-line–associated bloodstream infections (CLABSIs), catheter-associated urinary tract infections (CAUTIs), ventilator-associated events (VAEs), select surgical site infections (SSIs), laboratory-identified (LabID) methicillin-resistant *Staphylococcus aureus* (MRSA) bacteremia, and LabID *Clostridioides difficile* (CDI) events as applicable.^5–7^ The NHSN uses standard surveillance protocols to define and collect data from hospitals for these HAIs.^5^ Given the reporting deadline for the Centers for Medicare and Medicaid Services (CMS) Hospital-Acquired Conditions Reduction Program (HACRP), data for 2021-Q4 were not fully reported to NHSN at the time of this analysis and thus were not included.^8^

The 2021 quarterly results were compared to the same quarters from 2019; this comparison was restricted to hospitals that reported complete data for both quarters in each comparison (and for the same locations when applicable). Analyses were limited to the units and procedures required under HACRP where applicable.^8^ The percentage change in SIR or in SUR was calculated for each quarterly comparison. A 2-tailed *P* <.05 calculated using a mid-P exact test was considered statistically significant. Data were analyzed using SAS version 9.4 software (SAS Institute, Cary, NC).

## Results

### First quarter 2021 (2021-Q1)

During 2021-Q1, SIRs were significantly higher than SIRs during the prepandemic period (2019-Q1) for CLABSI, CAUTI, VAE, and MRSA bacteremia (Table 1). The highest SIRs occurred for VAE (SIR, 1.43) and MRSA bacteremia (SIR, 1.17), showing a 51% and 39% increase above 2019-Q1, respectively. In addition, device utilization was significantly higher during 2021-Q1 compared with the prepandemic period for all 3 devices measured; the greatest increase (32%) in SUR occurred for ventilators (Supplementary Material online).

**Table 1.** Changes in National Healthcare-Associated Infection (HAI) Standardized Infection Ratios (SIRs) Between 2021 Quarter 1 (2021-Q1) and 2019 Quarter 1 (2019-Q1)

HAI Type	No. of Hospitals^a^	% Change in SIR^b^	95% CI	2021-Q1				2019-Q1			
				No. of HAIs Reported	No. of HAIs Predicted	Device/Patient Days or Procedures^c^	SIR	No. of HAIs Reported	No. of HAIs Predicted	Device/Patient Days or Procedures^c^	SIR
CLABSI^e^	3,394	45.3^d^	(38.6 to 52.2)	4,505	4,515.37	4,489,151	0.998	2,924	4,256.41	4,244,791	0.687
CAUTI^f^	3,389	11.5^d^	(6.7 to 16.6)	4,318	5,174.38	4,429,547	0.834	3,593	4,801.64	4,125,335	0.748
VAE^g^	1,440	50.9^d^	(42.5 to 52.5)	10,270	7,176.85	1,055,497	1.431	5,047	5,322.98	764,599	0.948
SSI, colon surgery^h^	2,776	−5.3	(−11.3 to 1.3)	1,693	2,063.48	78,726	0.820	1,805	2,085.28	82,523	0.866
SSI, abdominal hysterectomy^h^	2,424	5.4	(−7.5 to 20.2)	429	439.33	63,251	0.976	468	505.40	77,481	0.926
LabID MRSA bacteremia^i^	3,477	39.2^d^	(31.7 to 47.2)	2,999	2,573.38	39,791,227	1.165	2,087	2,493.46	40,214,206	0.837
LabID CDI^j^	3,476	−15.6^d^	(−17.6 to −13.5)	11,534	21,765.23	37,024,548	0.530	15,061	23,996.48	37,149,384	0.628

Note. HAI, healthcare-associated infection; CI, confidence interval; CLABSI, central-line–associated bloodstream infection; CAUTI, catheter-associated urinary tract infection; VAE, ventilator-associated event; SSI, surgical site infection; LabID, laboratory-identified; MRSA, methicillin-resistant *Staphylococcus aureus*; CDI, *Clostridioides difficile* infection; CMS, Centers for Medicare and Medicaid Services; ICU, intensive care unit; NHSN, National Healthcare Safety Network. Data as of September 1, 2021.

a The number of acute-care hospitals that reported complete HAI surveillance data for both quarters in the comparison and for the same location when applicable. SSI hospital counts represent those hospitals that reported procedure-level data eligible for inclusion in the adult Complex Admission-Readmission models used for SSI SIR calculations. Hospitals that performed zero procedures, or had zero procedures included in the SIR calculation, were excluded from the SSI hospital counts.

b % change was calculated as follows: [(2021 SIR/2019 SIR) − 1)] × 100. This formula is equivalent to [(2021 SIR − 2019 SIR) ÷ 2019 SIR] × 100. This value is reported regardless of statistical significance, and a non-significant value should be interpreted as no statistical difference between the 2021 and 2019 SIRs.

c Device days are shown for CLABSI, CAUTI, and VAE. Procedure counts are shown for SSI. Patient days are shown for LabID events.

d Statistically significant result, as indicated by 2-tailed *P* ≤ 0.05 and the 95% CI not including zero.

e CLABSI SIRs were calculated using data from adult and pediatric ICUs, neonatal ICUs, and adult and pediatric medical, surgical, and medical–surgical wards.

f CAUTI SIRs were calculated using data from adult and pediatric ICUs, and adult and pediatric medical, surgical, and medical–surgical wards.

g VAE SIRs were calculated using data from adult ICUs and wards.

h SSIs included were those classified as deep incisional or organ-space infections following adult inpatient procedures and were detected during the same admission as the surgical procedure or upon readmission to the same hospital. The NHSN Complex Admission–Readmission model was used for SIR calculations.

i MRSA bacteremia SIRs were calculated using data from all inpatient locations in the hospital (facility-wide inpatient, or FacWideIN) except inpatient rehabilitation and inpatient psychiatric units certified by the CMS. The number of reported and predicted HAIs were limited to hospital-onset events.

j CDI SIRs were calculated using data from all inpatient locations in the hospital (FacWideIN) except neonatal ICUs, newborn nurseries, and inpatient rehabilitation and inpatient psychiatric units certified by the CMS. The number of reported and predicted HAIs were limited to hospital-onset incident events.

### Second quarter 2021 (2021-Q2)

**Fig. 1.**
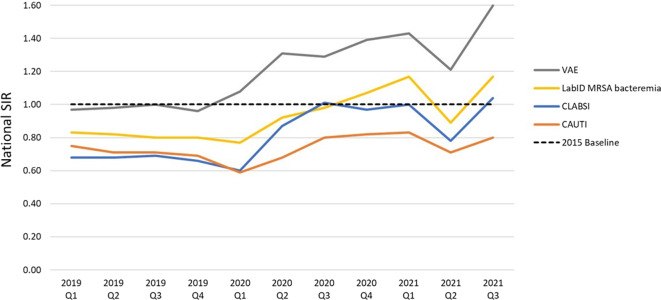
Quarterly national SIRs for select HAI types, 2019-Q1 through 2021-Q3. The HAIs shown on this graph have been most affected by the COVID-19 pandemic, as demonstrated by CDC data.^1,2^ SIRs for other types of infections are available in Tables 1–3 and in prior reports.^1,2^ This graph displays the quarterly SIR point estimates from 2019-Q1 through 2021-Q3 and does not constitute a statistical trend analysis. Note: SIR, standardized infection ratio; HAI, healthcare-associated infection; VAE, ventilator-associated event; LabID, laboratory–identified; MRSA, methicillin-resistant *Staphylococcus aureus*; CLABSI, central-line–associated bloodstream infection; CAUTI, catheter-associated urinary tract infection.

The SIRs for CLABSI (SIR, 0.78), VAE (SIR, 1.21), and MRSA bacteremia (0.89) reached their lowest values since the start of the pandemic in 2021-Q2 (Fig. 1). Additionally, the number of national ventilator days decreased by almost 20% between 2021-Q1 (n = 1,055,497) and 2021-Q2 (n = 849,062). Although encouraging results were seen during this time, SIRs for CLABSI, VAE, and MRSA bacteremia, and device utilization for all 3 devices remained significantly higher than prepandemic values (Table 2 and Supplementary Material online).

**Table 2.** Changes in National Healthcare-Associated Infection (HAI) Standardized Infection Ratios (SIRs) Between 2021 Quarter 2 (2021-Q2) and 2019 Quarter 2 (2019-Q2)

HAI Type	No. of Hospitals^a^	% Change in SIR^b^	95% CI	2021-Q2				2019-Q2			
				No. of HAIs Reported	No. of HAIs Predicted	Device/Patient Days or Procedures^c^	SIR	No. of HAIs Reported	No. of HAIs Predicted	Device/Patient Days or Procedures^c^	SIR
CLABSI^e^	3,398	14.6^d^	(9.0 to 20.5)	3,303	4,244.28	4,186,378	0.778	2,798	4,120.19	4,090,639	0.679
CAUTI^f^	3,394	−0.4	(−5.1 to 4.5)	3,369	4,773.48	4,029,827	0.706	3,230	4,557.72	3,894,139	0.709
VAE^g^	1,411	26.3^d^	(22.1 to 31.4)	7,224	5,976.18	849,062	1.209	4,725	4,935.08	703,586	0.957
SSI, colon surgery^h^	2,770	−2.5	(−8.6 to 3.9)	1,879	2,217.10	84,831	0.848	1,844	2,119.91	83,625	0.870
SSI, abdominal hysterectomy^h^	2,443	0.8	(−10.6 to 13.8)	514	520.09	75,323	0.988	542	553.12	84,302	0.980
LabID MRSA bacteremia^i^	3,474	8.3^d^	(2.0 to 15.0)	2,262	2,540.52	39,783,230	0.890	2,021	2,457.78	39,166,111	0.822
LabID CDI^j^	3,473	−14.1^d^	(−16.3 to −12.0)	10,900	21,817.08	36,808,005	0.500	13,452	23,106.96	36,015,721	0.582

Note. HAI, healthcare-associated infection; CI, confidence interval; CLABSI, central–line–associated bloodstream infection; CAUTI, catheter-associated urinary tract infection; VAE, ventilator-associated event; SSI, surgical site infection; LabID, laboratory-identified; MRSA, methicillin-resistant *Staphylococcus aureus*; CDI, *Clostridioides difficile* infection; CMS, Centers for Medicare and Medicaid Services; ICU, intensive care unit; NHSN, National Healthcare Safety Network. Data as of December 1, 2021.

a The number of acute-care hospitals that reported complete HAI surveillance data for both quarters in the comparison and for the same location when applicable. SSI hospital counts represent those hospitals that reported procedure-level data eligible for inclusion in the adult Complex Admission-Readmission models used for SSI SIR calculations. Hospitals that performed zero procedures, or had zero procedures included in the SIR calculation, were excluded from the SSI hospital counts.

b % change was calculated as follows: [(2021 SIR/2019 SIR) − 1)] × 100. This formula is equivalent to [(2021 SIR − 2019 SIR) ÷ 2019 SIR] × 100. This value is reported regardless of statistical significance, and a non-significant value should be interpreted as no statistical difference between the 2021 and 2019 SIRs.

c Device days are shown for CLABSI, CAUTI, and VAE. Procedure counts are shown for SSI. Patient days are shown for LabID events.

d Statistically significant result, as indicated by 2-tailed *P* ≤ 0.05 and the 95% CI not including zero.

e CLABSI SIRs were calculated using data from adult and pediatric ICUs, neonatal ICUs, and adult and pediatric medical, surgical, and medical–surgical wards.

f CAUTI SIRs were calculated using data from adult and pediatric ICUs, and adult and pediatric medical, surgical, and medical–surgical wards.

g VAE SIRs were calculated using data from adult ICUs and wards.

h SSIs included were those classified as deep incisional or organ-space infections following adult inpatient procedures and were detected during the same admission as the surgical procedure or upon readmission to the same hospital. The NHSN Complex Admission–Readmission model was used for SIR calculations.

i MRSA bacteremia SIRs were calculated using data from all inpatient locations in the hospital (facility-wide inpatient, or FacWideIN) except inpatient rehabilitation and inpatient psychiatric units certified by the CMS. The number of reported and predicted HAIs were limited to hospital-onset events.

j CDI SIRs were calculated using data from all inpatient locations in the hospital (FacWideIN) except neonatal ICUs, newborn nurseries, and inpatient rehabilitation and inpatient psychiatric units certified by the CMS. The number of reported and predicted HAIs were limited to hospital-onset incident events.

### Third quarter 2021 (2021-Q3)

Despite decreases in 2021-Q2, a reversal in the direction of SIRs was observed in 2021-Q3. CLABSI, CAUTI, VAE, and MRSA bacteremia SIRs were significantly and substantially higher compared to the SIRs from the prior 2021 quarters and the corresponding prepandemic quarter (Table 3). Particularly, the CLABSI and VAE SIRs of 1.04 and 1.60, respectively, were higher in 2021-Q3 than during any previous quarter since the beginning of 2019. The number of VAEs during 2021-Q3 was 149% higher than the number reported during 2019-Q3 from the same set of hospitals, and the ventilator SUR increased by 40% from 2019-Q3 (SUR, 0.91) to 2021-Q3 (SUR, 1.28).

**Table 3.** Changes in National Healthcare-Associated Infection (HAI) Standardized Infection Ratios (SIRs) Between 2021 Quarter 3 (2021-Q3) and 2019 Quarter 3 (2019-Q3)

HAI Type	No. of Hospitals^a^	% Change in SIR^b^	95% CI	Preliminary 2021-Q3				2019-Q3			
				No. of HAIs Reported	No. of HAIs Predicted	Device/Patient Days or Procedures^c^	SIR	No. of HAIs Reported	No. of HAIs Predicted	Device/Patient Days or Procedures^c^	SIR
CLABSI^e^	3,215	48.4^d^	(41.6 to 55.5)	4,741	4,573.59	4,539,805	1.037	2,761	3,951.77	3,911,645	0.699
CAUTI^f^	3,208	13.6^d^	(8.4 to 19.1)	4,074	5,087.85	4,325,035	0.801	2,997	4,251.81	3,592,706	0.705
VAE^g^	1,343	60.2^d^	(54.8 to 65.8)	11,353	7,097.22	1,036,989	1.600	4,553	4,559.15	639,187	0.999
SSI, colon surgery^h^	2,593	−9.2^d^	(−15.2 to −2.8)	1,542	1,936.21	73,976	0.796	1,774	2,021.71	79,396	0.877
SSI, abdominal hysterectomy^h^	2,283	−4.1	(−15.5 to 8.7)	428	410.90	58,550	1.042	559	514.35	78,020	1.087
LabID MRSA bacteremia^i^	3,272	45.1^d^	(37.0 to 53.6)	3,066	2,625.60	40,987,396	1.168	1,927	2,393.81	37,865,802	0.805
LabID CDI^j^	3,269	−14.5^d^	(−16.7 to −12.2)	10,565	21,905.36	37,822,902	0.482	12,408	21,999.91	34,586,039	0.564

Note. HAI, healthcare-associated infection; CI, confidence interval; CLABSI, central–line–associated bloodstream infection; CAUTI, catheter-associated urinary tract infection; VAE, ventilator-associated event; SSI, surgical site infection; LabID, laboratory-identified; MRSA, methicillin-resistant *Staphylococcus aureus*; CDI, *Clostridioides difficile* infection; CMS, Centers for Medicare and Medicaid Services; ICU, intensive care unit; NHSN, National Healthcare Safety Network. Data as of January 1, 2022.

a The number of acute-care hospitals that reported complete HAI surveillance data for both quarters in the comparison and for the same location when applicable. SSI hospital counts represent those hospitals that reported procedure-level data eligible for inclusion in the adult Complex Admission-Readmission models used for SSI SIR calculations. Hospitals that performed zero procedures, or had zero procedures included in the SIR calculation, were excluded from the SSI hospital counts.

b % change was calculated as follows: [(2021 SIR/2019 SIR) − 1)] × 100. This formula is equivalent to [(2021 SIR − 2019 SIR) ÷ 2019 SIR] × 100. This value is reported regardless of statistical significance, and a non-significant value should be interpreted as no statistical difference between the 2021 and 2019 SIRs.

c Device days are shown for CLABSI, CAUTI, and VAE. Procedure counts are shown for SSI. Patient days are shown for LabID events.

d Statistically significant result, as indicated by 2-tailed *P* ≤ 0.05 and the 95% CI not including zero.

e CLABSI SIRs were calculated using data from adult and pediatric ICUs, neonatal ICUs, and adult and pediatric medical, surgical, and medical–surgical wards.

f CAUTI SIRs were calculated using data from adult and pediatric ICUs, and adult and pediatric medical, surgical, and medical–surgical wards.

g VAE SIRs were calculated using data from adult ICUs and wards.

h SSIs included were those classified as deep incisional or organ-space infections following adult inpatient procedures and were detected during the same admission as the surgical procedure or upon readmission to the same hospital. The NHSN Complex Admission–Readmission model was used for SIR calculations.

i MRSA bacteremia SIRs were calculated using data from all inpatient locations in the hospital (facility-wide inpatient, or FacWideIN) except inpatient rehabilitation and inpatient psychiatric units certified by the CMS. The number of reported and predicted HAIs were limited to hospital-onset events.

j CDI SIRs were calculated using data from all inpatient locations in the hospital (FacWideIN) except neonatal ICUs, newborn nurseries, and inpatient rehabilitation and inpatient psychiatric units certified by the CMS. The number of reported and predicted HAIs were limited to hospital-onset incident events.

### CDI and SSI

The 2021 CDI SIRs were significantly lower than those from 2019 for all quarters analyzed. Continued decreases in CDI were evident throughout 2021: the SIR for 2021-Q2 was 0.50 and the SIR for Q3 was 0.48, which were lower than those from the prior 2020 and 2021 quarters. For most quarters and procedure types analyzed, no significant changes in SSI incidence were detected.

## Discussion

Our analysis revealed elevated incidence of CLABSIs, CAUTIs, VAEs, and MRSA bacteremia infections during 2021, especially during the first and third quarters of the year.

During 2021-Q1, all-time highs of COVID-19–associated hospitalizations were recorded throughout the country.^9^ Although large increases were noted in CLABSI, VAE, and MRSA bacteremia in 2021-Q1, the increase in the CAUTI SIR was modest. Improvements in CLABSI, CAUTI, VAE, and MRSA bacteremia SIRs were observed in 2021-Q2, coincident with the dramatic reduction in nationwide COVID-19 hospitalizations.^9^ However, as the severe acute respiratory coronavirus virus 2 (SARS-CoV-2) δ (delta) variant emerged in 2021-Q3, dramatic increases in SIRs were observed again.^10^ Although data from the SARS-CoV-2 Ο (omicron) variant surge will be forthcoming, SIRs might follow similar trends in 2021-Q4 and early 2022.

Changes in most SIRs were driven by changes in the number of reported HAIs, with several factors contributing to such changes. First, device-associated HAIs were likely affected by the continued alteration of hospital practices that occurred throughout the pandemic. Modifications of CLABSI prevention practices during 2020 are well documented,^3,11^ and prevention practices likely continued to be altered during 2021. By contrast, the modest increase in CAUTI SIRs may be related to the fact that catheter removal, a primary approach to CAUTI prevention, was still possible even during times of stress on the healthcare system. Conversely, pandemic-related improvements in hand hygiene, PPE practices, and environmental cleaning may have contributed to the decreases observed in the CDI SIR. Colon surgeries and abdominal hysterectomies were not typically performed as part of COVID-19 care, and process flows in the operating room remained relatively unchanged during this time.^12^ This finding may explain the lack of significant changes observed in SSI SIRs.

Second, different patients may have been admitted to healthcare settings in 2021 compared to the prepandemic period, and the increases in SIRs may be explained by changes in the proportion of patients with different characteristics (eg, race or ethnicity and comorbidities). Although some characteristics (eg, patient location) were controlled for in the device-associated HAI SIRs, the risk-adjustment models may not have adjusted for all relevant characteristics. In addition, increases in SIRs could have been due to increased patient morbidity from COVID-19. One 2020 study found that the most common cause of VAEs during surges of COVID-19 was acute respiratory distress syndrome, whereas most VAEs in 2019 were caused by less severe events such as pneumonia.^13^ The national SIR for VAE increased the most of all HAIs in 2021, with the greatest increase (60%) occurring during the SARS-CoV-2 δ (delta) variant surge in 2021-Q3. Although a previous analysis found no change in the proportion of adults requiring ventilation during the SARS-CoV-2 δ (delta) variant surge compared to the first half of 2021,^14^ the largest increase (40%) in the national ventilator SUR since the start of the pandemic occurred during 2021-Q3. Overall increases in device-associated HAI SIRs, particularly VAE, may reflect an increase in the frequency and duration of device use and an increase in the average length of stay during COVID-19 surges.^1^

The limitations of this analysis are similar to those previously reported.^1^ The 2021-Q3 results were generated before the HACRP reporting deadline and should be considered preliminary. Hospitals and units that opened during 2020 or 2021 were not included. All HAIs regardless of patient’s COVID-19 status were included, and the impact of a COVID-19 diagnosis on the SIRs could not be determined.

For most HAIs, our results are representative of most acute-care hospitals in the United States and provide a national picture of the impact of COVID-19 on HAI incidence. Our findings describe the increases in HAIs that occurred during the 2021 COVID-19 pandemic year and underscore the continued challenges experienced in infection prevention. Resilient approaches are needed to reduce HAIs in 2022 and beyond.^15^

## Supplementary material

For supplementary material accompanying this paper visit https://doi.org/10.1017/ice.2022.116. click here to view supplementary material
