# Supplementary material for: Continued increases in the incidence of healthcare-associated infection (HAI) during the second year of the coronavirus disease 2019 (COVID-19) pandemic
Source: Infect Control Hosp Epidemiol. 2022 May 20:1–5. doi: 10.1017/ice.2022.116 (PMC9237489; doi:10.1017/ice.2022.116)
Supplement: Supplementary file 1 [file S0899823X22001167sup001.docx]

**Continued Increases in HAI Incidence During the Second Year of the COVID-19 Pandemic**

Supplemental Materials: Changes in national device standardized utilization ratios (SURs) between 2021 and 2019, by quarter

|  |  |  |  | **2021 Q1** | | | **2019 Q1** | | |
| --- | --- | --- | --- | --- | --- | --- | --- | --- | --- |
| **Device Type** | **# hospitals^a^** | **% change in SUR^b^** | **95% CI around   % change** | **# observed device days** | **# predicted device days** | **SUR** | **# observed device days** | **# predicted device days** | **SUR** |
| Central line^d^ | 3,394 | 6.5^c^ | (6.4, 6.7) | 4,489,151 | 4,826,132.82 | 0.930 | 4,244,791 | 4,862,573.44 | 0.873 |
| Urinary Catheter^e^ | 3,389 | 7.0^c^ | (6.7, 7.0) | 4,429,547 | 4,891,599.16 | 0.906 | 4,125,335 | 4,869,269.48 | 0.847 |
| Ventilator^f^ | 1,440 | 31.7^c^ | (31.3, 32.1) | 1,055,497 | 807,248.38 | 1.308 | 764,599 | 770,070.60 | 0.993 |
|  |  |  |  |  |  |  |  |  |  |
|  |  |  |  | **2021 Q2** | | | **2019 Q2** | | |
| **Device Type** | **# hospitals^a^** | **% change in SUR^b^** | **95% CI around   % change** | **# observed device days** | **# predicted device days** | **SUR** | **# observed device days** | **# predicted device days** | **SUR** |
| Central line^d^ | 3,398 | 1.0^c^ | (0.90, 1.2) | 4,186,378 | 4,793,035.10 | 0.873 | 4,090,639 | 4,732,499.88 | 0.864 |
| Urinary Catheter^e^ | 3,394 | 2.1^c^ | (2.0, 2.3) | 4,029,827 | 4,772,694.51 | 0.844 | 3,894,139 | 4,710,750.79 | 0.827 |
| Ventilator^f^ | 1,411 | 16.5^c^ | (16.1, 16.8) | 849,062 | 767,066.42 | 1.107 | 703,586 | 740,269.52 | 0.950 |
|  |  |  |  |  |  |  |  |  |  |
|  |  |  |  | **Preliminary 2021 Q3** | | | **2019 Q3** | | |
| **Device Type** | **# hospitals^a^** | **% change in SUR^b^** | **95% CI around   % change** | **# observed device days** | **# predicted device days** | **SUR** | **# observed device days** | **# predicted device days** | **SUR** |
| Central line^d^ | 3,215 | 6.2^c^ | (6.1, 6.4) | 4,539,805 | 4,982,228.26 | 0.911 | 3,911,645 | 4,559,457.40 | 0.858 |
| Urinary Catheter^e^ | 3,208 | 8.4^c^ | (8.2, 8.5) | 4,325,035 | 4,993,980.87 | 0.866 | 3,592,706 | 4,494,354.93 | 0.799 |
| Ventilator^f^ | 1,343 | 40.2^c^ | (39.7, 40.5) | 1,036,989 | 812,310.71 | 1.277 | 639,187 | 701,497.99 | 0.911 |

**Note**: CI, confidence interval; ICU, intensive care unit. Each quarter's comparison uses data that were frozen at the following points in time: Quarter-1 data as of September 1, 2021; Quarter-2 data as of December 1, 2021; Quarter-3 data as of January 1, 2022.

**Footnotes, Supplemental Materials:**

**^a^** The number of acute care hospitals that reported complete HAI surveillance data for the same location for both quarters in the comparison.

**^b^** % change was calculated as follows: [(2021 SUR/2019 SUR) − 1)] × 100. This formula is equivalent to: [(2021 SUR − 2019 SUR) ÷ 2019 SUR] × 100.

**^c^** Statistically significant result, as indicated by 2-tailed *P* ≤ 0.05 and the 95% CI not including the value of zero.

**^d^** Central line SURs were calculated using data from adult and pediatric ICUs, neonatal ICUs, and adult and pediatric medical, surgical, and medical–surgical wards.

**^e^** Urinary catheter SURs were calculated using data from adult and pediatric ICUs, and adult and pediatric medical, surgical, and medical–surgical wards.

**^f^**Ventilator SURs were calculated using data from adult ICUs and wards.
